# Among the Colleges

**Published:** 1899-11

**Authors:** 


### AMONG THE COLLEGES.

The New York State Veterinary College has booked a class of sixteen freshmen for this year's college work, an increase of 100 per cent. over the year preceding. Those directing the school's interest feel much encouraged as to the outcome of so advanced a curriculum and high standard of entrance requirements.

Dr. John R. Mohler, of the Bureau of Animal Industry, will lecture on meat-inspection and parasitology at this session of the United States College of Veterinary Surgeons.

Prof. Charles F. Dawson, formerly of the Veterinary Department of the Columbian University, will deliver a course of lectures on general pathology to the students of the United States College of Veterinary Surgeons.

